# Validation of droplet digital PCR for cytokeratin 19 mRNA detection in canine peripheral blood and mammary gland

**DOI:** 10.1038/s41598-022-17493-5

**Published:** 2022-08-10

**Authors:** Potsawat Tanvetthayanont, Teerapong Yata, Jiranun Boonnil, Sasithon Temisak, Suppawiwat Ponglowhapan

**Affiliations:** 1 Department of Obstetrics Gynaecology and Reproduction, Faculty of Veterinary Science, Chulalongkorn University, Bangkok, 10330 Thailand; 2 Unit of Biochemistry, Department of Physiology, Faculty of Veterinary Science, Chulalongkorn University, Bangkok, 10330 Thailand; 3 National Institute of Metrology (NIMT), Pathumthani, 12120 Thailand

**Keywords:** Oncology, Cancer

## Abstract

In humans, peripheral blood cytokeratin 19 (CK19) mRNA-positive circulating tumor cells (CTCs) was utilized to identify early-stage breast cancer patients with micrometastatic disease who are at risk for disease progression and monitor treatment response in patients with advanced disease. To our knowledge, there has been little research regarding CK19 in canine mammary tumors (CMTs) using molecular methods. A droplet digital PCR (ddPCR) is proposed as a precise and sensitive quantification of nucleic acid targets. Hence, this study aimed to validate a newly designed assay for CK19 detection in canine blood and mammary tissue, along with the reference gene HPRT, by ddPCR. All primers and probes showed a precise match with the exon region of target genes. The assay exhibited PCR efficacy of 90.4% and 91.0% for CK19 and HPRT amplifications with linearity, respectively. The annealing temperature (T_a_) for duplex ddPCR was 55 °C, providing the highest concentrations of both genes tested by the synthetic plasmid DNA. The limit of detection (LOD) of CK19 and HPRT were 2.16 ± 1.27 and 2.44 ± 1.31 copies/µL, respectively. Finally, the ddPCR assay was validated with canine peripheral blood, non-neoplastic mammary tissues and spiked samples. Our findings provide a new platform for CK19 studies in CMT diagnosis through blood and mammary tissues.

## Introduction

Canine mammary tumors (CMTs) are one of the most common neoplasia (28%) in female dogs, and approximately half of CMTs are malignant, which is a substantial health problem in this population^1,2^. CMTs are a group of tumors that are heterogeneous in terms of morphology and biological behavior, and share similarities with the same disease in humans^3,4^. Cytokeratin 19 (CK19) is a protein belonging to complex intracytoplasmic cytoskeleton that can be found in ductal epithelial cells. Previous studies on mammary tumors in humans^5^ and dogs^6^ found that CK19 negative immunohistochemistry was associated with a high histological grade, lympho-vascular invasion and high Ki-67 index, and were estrogen receptor negative. Although excisional biopsy, sometimes combined with immunohistochemistry, is used as a routine diagnosis and treatment for CMTs, unfortunately they cannot identify early-stage breast cancer patients with micrometastatic disease. Currently, liquid biopsy is a new diagnostic method which uses blood to detect micrometastatic tumor cells^7–11^. Circulating tumor cells (CTCs) detected by CK19 mRNA positive can be found in peripheral blood samples of breast cancer patients, and there are correlations between circulating CK19 mRNA positive and poor prognosis^7–11^. Unfortunately, there is no study associated with CK19 detection in canine peripheral blood for CMT diagnosis.

In recent times, droplet digital PCR (ddPCR) is a new technology providing an absolute and precise quantification of target gene, with no requirement of a standard curve. ddPCR is based on water–oil emulsion droplet technology. A sample is fractionated into up to approximately 20,000 (nanoliter-sized) droplets, and PCR amplification of the template molecules occurs in each individual droplet. Following PCR, each droplet is analyzed or read to determine the fraction of PCR-positive droplets in the original sample. The fraction of positive droplets is then fitted to a Poisson distribution to determine the absolute initial copy number of the target DNA molecule, reported as copies/µL^12,13^. In the field of molecular oncology, many studies have demonstrated a high degree of sensitivity and precision of ddPCR, compared to quantitative real-time PCR (qPCR)^12–15^. Moreover, this technology has been applied to investigate the expression of genes in tumor tissue samples such as breast cancer and gastric cancer in humans^16–19^. Nevertheless, the use of ddPCR in veterinary oncology is limited, and there has been no study on CK19 mRNA detection in CMTs using ddPCR. To investigate this, a validation method for the gene detection is required. The objectives of this study were to develop and validate the assays for CK19 mRNA detection, along with the HPRT reference gene, by ddPCR in canine peripheral blood and mammary tissues.

## Methods

### Sample collections

The samples were canine peripheral blood (n = 3) and non-neoplastic mammary tissues (n = 3) obtained from the dogs that were presented to the Division of Obstetrics, Gynaecology and Reproduction, Small Animal Teaching Hospital, Faculty of Veterinary Science, Chulalongkorn University between January and March 2021. The peripheral blood (5 mL) was collected from clinically healthy dogs based on screening tests including physical examination, hydration status assessment, CBC, serum biochemistry (alanine aminotransferase and creatinine) and diagnostic imaging (thoracic radiography and abdominal ultrasonography). The non-neoplastic mammary tissues (50 mg) were collected from non-tumor mammary glands. The tissue was histopathologically examined to assure that there were no evidences of cancerous cells. Blood and tissue samples were stored immediately at 4 °C. Thereafter, the process of RNA extraction was performed within 30–60 min. Synthetic plasmid DNA containing CK19 and HPRT was used as a positive template (Invitrogen, Massachusetts, United States) (see Supplementary Fig. S1).

### RNA extraction

Blood of a healthy female dog (5 mL) was added in the ratio of 20 mL of 1X RBC lysis buffer (Cell Signaling Technology, Danvers, Morocco) to 1 mL of blood in a 50 mL centrifuge tube, then centrifuged at 10,000 × *g* for 5 min at 4 °C, and the supernatant was then removed without disturbing the cell pellet. Meanwhile, 50 mg of non-neoplastic canine mammary tissue was lysed and homogenized with tissue homogenizer (FastPrep-24 5G, MP biomedicals, California, United States). The mRNA from blood sample (cell pellet) and homogenized tissue were extracted using TRIzol reagent (ThermoFisher Scientific, Massachusetts, United States), following the protocol described in the kit description. Finally, the RNA pellets were resuspended in 30 µL of RNase-free water and incubated in a heat block set at 55 °C for 10 min. The RNA was stored at − 80 °C until used. The RNA concentration was determined by a spectrophotometer (NanoDrop ND-1000; Peqlab, Erlangen, Germany) reading at 260 and 280 nm. The samples with a ratio of 1.8–2.0 (260/280) were included in the study. The RNA integrity was measured by a bioanalyzer (RNA600 Nano Chip) (Bioanalyzer2100, Agilent, California, United States). The RNA integrity number was above 5^20^. DNA contamination was then treated with DNase I (TURBO DNase, Invitrogen, Massachusetts, United States), including 200 ng of total nucleic acid solution, 1 × buffer (10 × TURBO DNase Buffer) in the RNA sample, 2 U of DNase I and DEPC-treated water to 50 µL final volume. The reaction was incubated at 37 °C for 30 min, and then EDTA was added to a final concentration of 15 mM over 10 min at 75 °C to stop the reaction.

### cDNA synthesis

Total RNA (1 µg) was reverse transcribed using a cDNA synthesis Kit (iScript Reverse Transcription Supermix Kit, California, United States). The reaction contained 4 µL of iScript RT Supermix, 1 µg of RNA template and nuclease-free water, used to dilute the total volume to 20 µL. The reactions were incubated at 25 °C for 5 min, 46 °C for 20 min and 95 °C for 1 min. No reverse transcriptase (no-RT) controls were also performed as described in the kit. The success of the cDNA synthesis was confirmed by PCR amplification of the canine housekeeping gene (HPRT). The cDNA was stored at − 80 °C until used.

### Primer and probe design

This section describes the design of the primers and probes for CK19 and HPRT, using the *Canis lupus familiaris* keratin 19 (CK19) mRNA (NM_001253742.1) and the *Canis familiaris* HPRT mRNA (AY283372.1), respectively. All primers and probes were designed by Primer Express Software Version 3.0.1 (ThermoFisher Scientific, Massachusetts, United States) with default settings, which follow the primer and probe design criteria^21^. The primers and hydrolysis probes for CK19 and HPRT were synthesized and purified by Macrogen Inc. (Seoul, Korea) (Table 1) and the locations of both primers are shown in Supplementary Figs. S2 and S3 (reference genome: CanFam3.1). To check the specificity of the primers and probes, the Primer-BLAST^22^ and BLAST^23^ were performed.

**Table 1:** Sequences of design primers and hydrolysis probes.

Gene	Accession number	5′-sequence-3′	Product size in base pairs	Genomic location (CanFam3.1 GCF_000002285.3)
CK19	NM_001253742.1	Forward CCAAGATTGTCCTGCAGATTGAC Reverse GCCTGCTCCGTCTCAAACTT Probe FAM-CCCGTCTGGCTGCGGATGACTTC-BHQ1	76	NC_006591.3 (21240778..21245039)
HPRT	AY283372.1	Forward CGAGATGTGATGAAGGAAATGG Reverse AGCAGGTCAGCAAAGAATTTATAGC Probe Hex-CCATCACATCGTAGCCCTCTGTGTGC-BHQ1	86	NC_006621.3 (105115732..105153702)

### Quantitative real-time PCR reaction

Quantitative real-time PCR (qPCR) method was used first to test the assays before moving to the ddPCR platform. The synthetic plasmid DNA templates were used to test the assay and PCR efficacy. The concentration of this plasmid consists of 10^0^–10^5^ copies of plasmid DNA templates. To test the assay with RNA from one peripheral blood sample and one non-neoplastic mammary tissue, a ten-fold serial dilution of the cDNA from blood and non-neoplastic mammary tissue was made up with nuclease-free water, creating a solution range of no-dilution to 1:1000.

The 20 µL qPCR mixture consisted of 10 µL Luna Universal qPCR Master Mix (BioRad, California, United States), 2.0 µL of DNA template, 300 nmol/L forward and reverse primers and 200 nmol/L probes. Three replicates of PCR were run to end point using a thermal cycler (CFX Connect Real-time PCR Detection System, BioRad, California, United States). The setting for PCR reaction was 1 cycle of 95 °C for 10 min, 45 cycles of 95 °C for 15 s and 45 cycles of 60 °C for 30 s. No template control (NTC) was also included.

### Droplet digital PCR reaction and the optimization of annealing temperature (T_***a***_)

The PCR protocols followed the MIQE guideline^24^ (see Supplementary Table S1). For optimization of T_a_, 10^3^ copies of the synthetic plasmid DNA were used as the template, and the gradient temperature between 55 and 65 °C was determined. HindIII (5 U) (New England Biolabs, Massachusetts, United States) was added into the ddPCR reaction to digest a recognition site between the target sequences. There were 2 types of ddPCR assays, including duplex and singleplex. In the duplex assay, both CK19 and HPRT assays were added into the PCR reaction, while in the singleplex ddPCR only CK19 assay or HPRT assay was included in the PCR mixture.

The 20 µL of ddPCR mixture consisted of 10 µL 2 × ddPCR Supermix for Probes (BioRad, California, United States), 2.5 µL of the template DNA samples, 300 nmol/L of forward and reverse primers and 250 nmol/L probes. NTCs also were performed. The ddPCR mixture was loaded into ddPCR 96-well Plates (BioRad, California, United States), mixed and centrifuged for 30 s at 1000 rpm. The plate was placed into the Automated Droplet Generator (AutoDG, BioRad, California, United States) to partition the sample into droplets, which combined oil (Automated Droplet Generation Oil for Probes, BioRad, California, United States) and sample. Next, a 96-well PCR plate containing droplets was sealed with foil by a PX PCR Plate Sealer (BioRad, California, United States). PCR was run to end point using a thermal cycler (T100 Thermal Cycler, BioRad, California, United States). The PCR cycle of ddPCR was performed at 95 °C for 10 min, 40 cycles of 94 °C for 30 s, 1 min at optimal temperature for annealing and extension, and 1 cycle of 98 °C for 10 min.

The droplets were read by a droplet reader (QX200 Droplet Reader, BioRad, California, United States). The droplet reader read each droplet to qualify droplets with a target or without. Data was analyzed with QuantaSoft Software v.1.7.4.0917 (BioRad, California, United States). The quantification measurements of the target molecule were presented as the copies/µL of sample.

### Limit of detection (LOD)

The LOD is defined as the lowest PCR copy number concentration that can be discriminated from zero with a level of confidence of 95%, where at least 95% of the replicates are positive, and a minimum of ten replicates should be performed^25^. To establish the measurement range, the synthetic plasmid DNA was prepared in the concentration range of 50,000–5 copies/µL in three replicates and used as the templates in ddPCR reactions. The copy number of the plasmid DNA were converted from ng obtained from a spectrophotometer (NanoDrop ND-1000; Peqlab, Erlangen, Germany). Then, to determine the LOD, the lowest concentration of the synthetic plasmid was measured by ddPCR with ten replicates.

### CK19 detection in peripheral blood and mammary tissue by ddPCR

The cDNA from canine peripheral blood (n = 3) and non-neoplastic mammary tissues (n = 3) was performed under the aforementioned ddPCR conditions for detection of CK19 and HPRT. To verify the method, 50 mg of canine malignant mammary tumor tissue (Tubulopapillary carcinoma) was performed a twofold weight reduction (50–1.56 mg) and then spiked into 5 mL of blood from a healthy female dog as positive controls. All samples were done in three replicates for the ddPCR method.

### Statistical analysis

Descriptive statistics were used to describe the copy numbers of CK19 and HPRT. The copy numbers derived from replicates were presented as means and standard deviations.

### Ethical approval

All procedures involving the handling and collection of samples were approved by the Institutional Animal Care and Use Committee of Chulalongkorn University (CU-IACUC approval number 2131037) and performed in accordance with relevant guidelines and regulations. Informed consent and permissions were obtained from all the dog owners. This study was carried out in compliance with the ARRIVE guidelines.

## Results

As the CK19 and HPRT sequences were different between dogs and humans (see Supplementary Figs. S4 and S5), in this study the primers of both CK19 and HPRT were newly designed precisely to the exon region of canine mRNA (see Supplementary Figs. S6 and S7). Hence, these assays can be used for detecting CK19 gene expression in dogs. The assays were blasted against the NCBI database and the probes were specific 100% match rate to *Canis lupus familiaris* keratin 19 mRNA and *Canis familiaris* HPRT mRNA, respectively (see Supplementary Figs. S8 and S9). The assays were tested with qPCR using the synthetic plasmid DNA template. The results showed that the assays generated PCR amplification curves of CK19 and HPRT (see Supplementary Fig. S10) with PCR efficacy 90.4% (R^2^ = 0.994, slope = − 3.58) for the CK19 assay and 91.0% (R^2^ = 0.993, slope = − 3.56) for the HPRT assay (see Supplementary Fig. S11). The PCR efficiency calculated from efficiency was 10^−1/slope−1^, where the slope was obtained from the linear regression. Moreover, cDNA from canine blood and non-neoplastic mammary tissue, were used as the template. The RIN of all mRNAs was above 5 (see Supplementary Fig. S12). The results showed that blood and non-neoplastic mammary tissue samples can also generate PCR amplification curves for HPRT (see Supplementary Fig. S13). However, CK19 amplification curve was observed only in the tissue sample, there was no CK19 amplification curve in the blood sample (see Supplementary Fig. S14).

**Figure 1:**
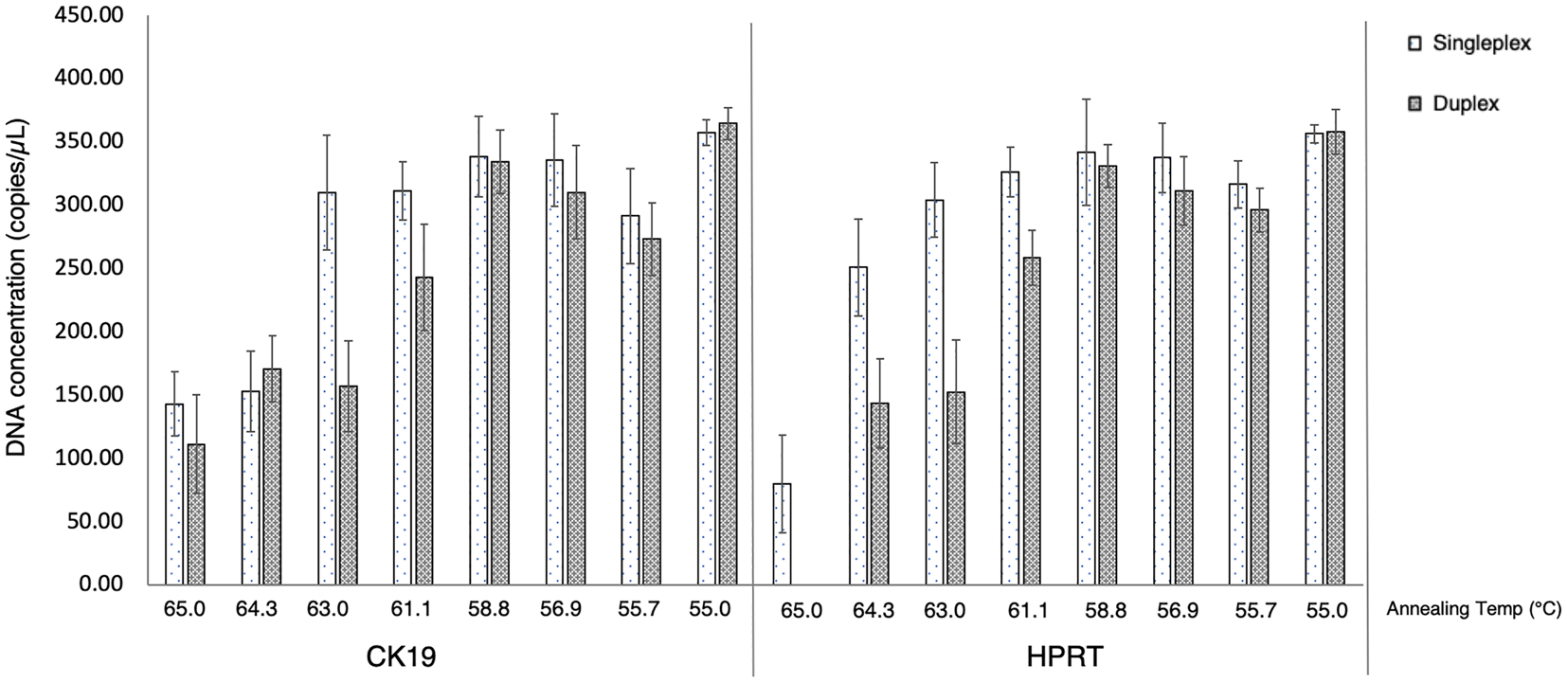
Comparison between singleplex and duplex ddPCR assays in quantification of CK19 and HPRT genes. The singleplex and duplex ddPCR assays measured the target genes across the different annealing temperatures.

**Figure 2:**
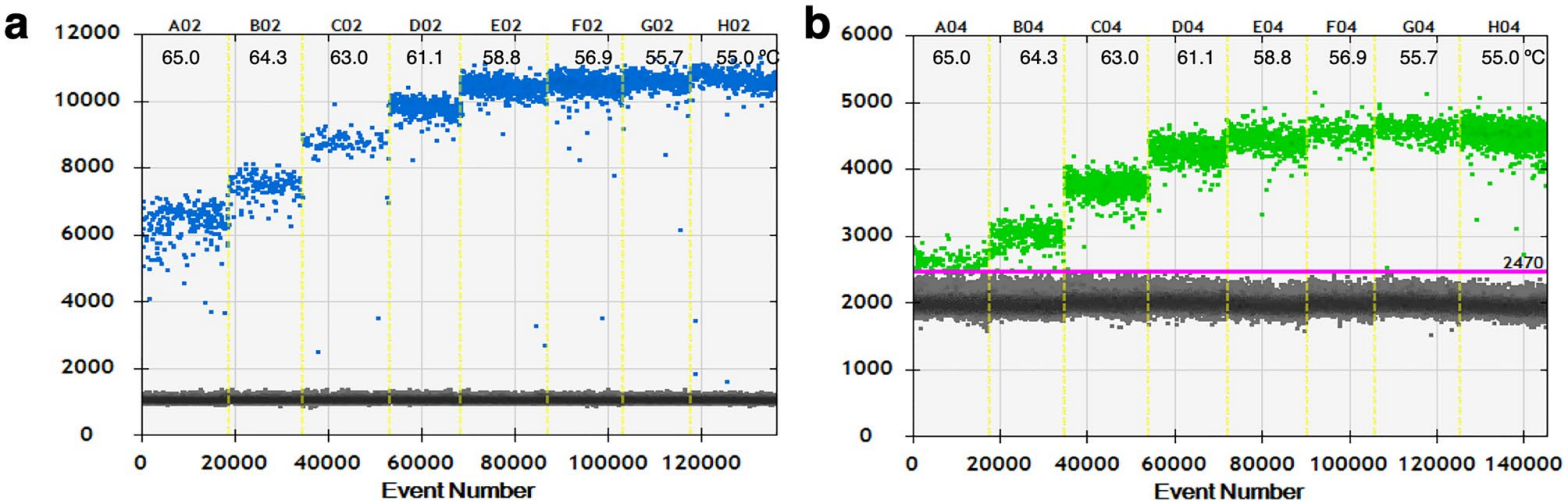
Singleplex ddPCR assays at different annealing temperatures for CK19 and HPRT detection. A thermal gradient PCR, ranging from 55 to 65 °C, showed in the 1D amplitude ddPCR plot of CK19 in FAM-Channel (**a**) and HPRT in HEX-Channel (**b**). Black dot populations are negative droplets, blue dot populations are positive droplets for CK19 and green dot populations are positive droplets for HPRT.

**Figure 3:**
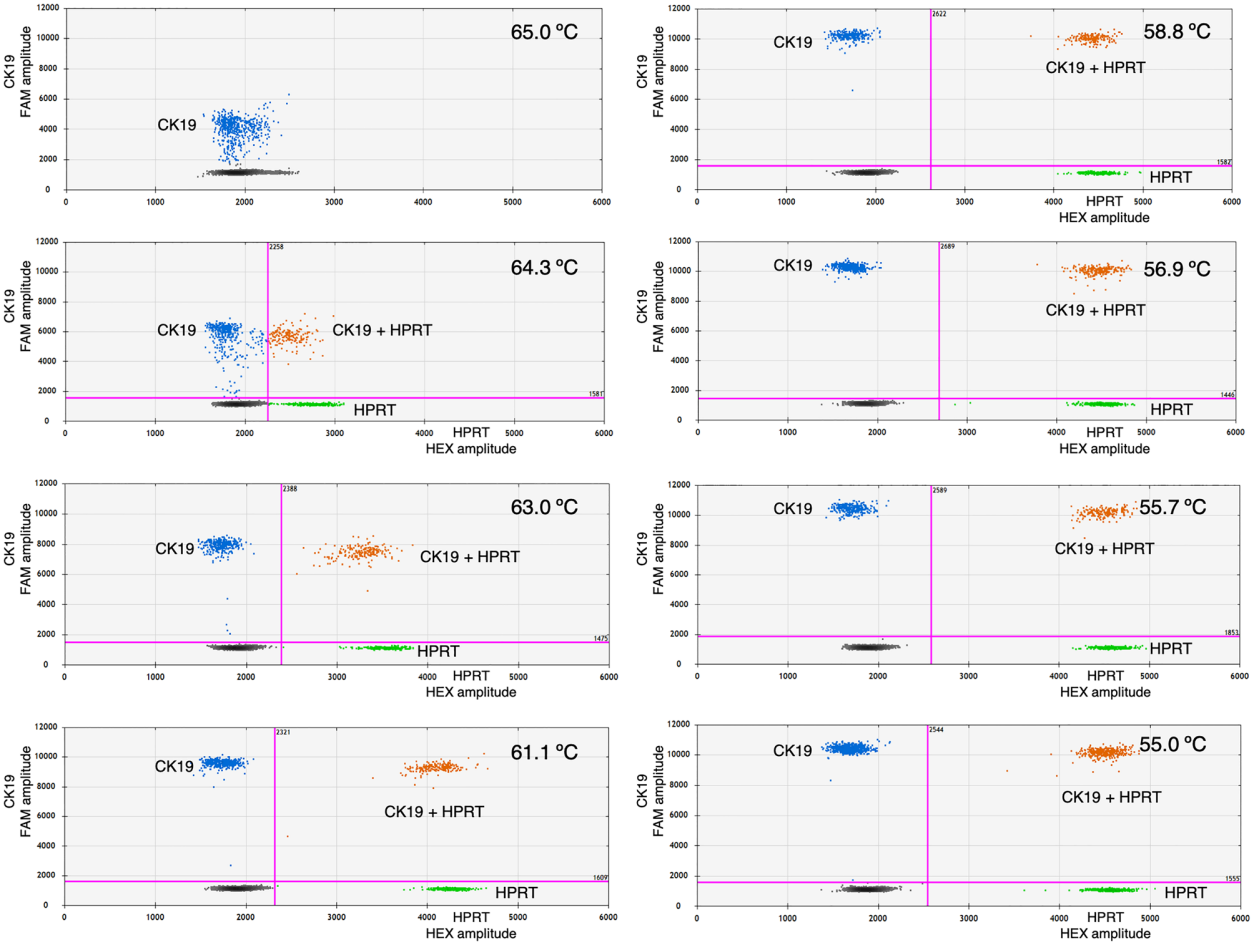
Duplex ddPCR assays in optimization of the annealing temperature for CK19 and HPRT detection. A thermal gradient PCR, ranging from 55 to 65 °C, showed in the 2D amplitude plot of CK19 in FAM-Channel, and HPRT in HEX-Channel. Black dot populations are negative droplets, blue dot populations are positive droplets for CK19, green dot populations are positive droplets for HPRT and orange dot populations are positive droplets for both genes.

Next, the assays were moved to the ddPCR platform. As the ddPCR method is more precise and sensitive than qPCR, ddPCR was proposed in this study for absolute quantification and detection of small amounts of target DNA. The synthetic plasmid DNA was used as the template, to optimize the T_a_ and for comparison between singleplex and duplex ddPCR assays. The results showed that at 55 °C, in both singleplex and duplex assays, the highest concentrations of CK19 and HPRT genes were provided, with no significant difference in concentrations between singleplex and duplex ddPCR assays (Fig. 1). Moreover, at this temperature (55 °C), positive droplets were clearly discriminated from negative ones in both singleplex (Fig. 2) and duplex ddPCR assays (Fig. 3). Therefore, 55 °C was used as the optimal T_a_ for the ddPCR method to detect CK19 and HPRT. As there were no differences in quantification of CK19 and HPRT between singleplex and duplex ddPCR assays, the duplex assay was selected for further experiments in simultaneous detection of CK19 and HPRT, due to concerns regarding consumable cost, time consumption and technical errors.

### LOD

**Figure 4:**
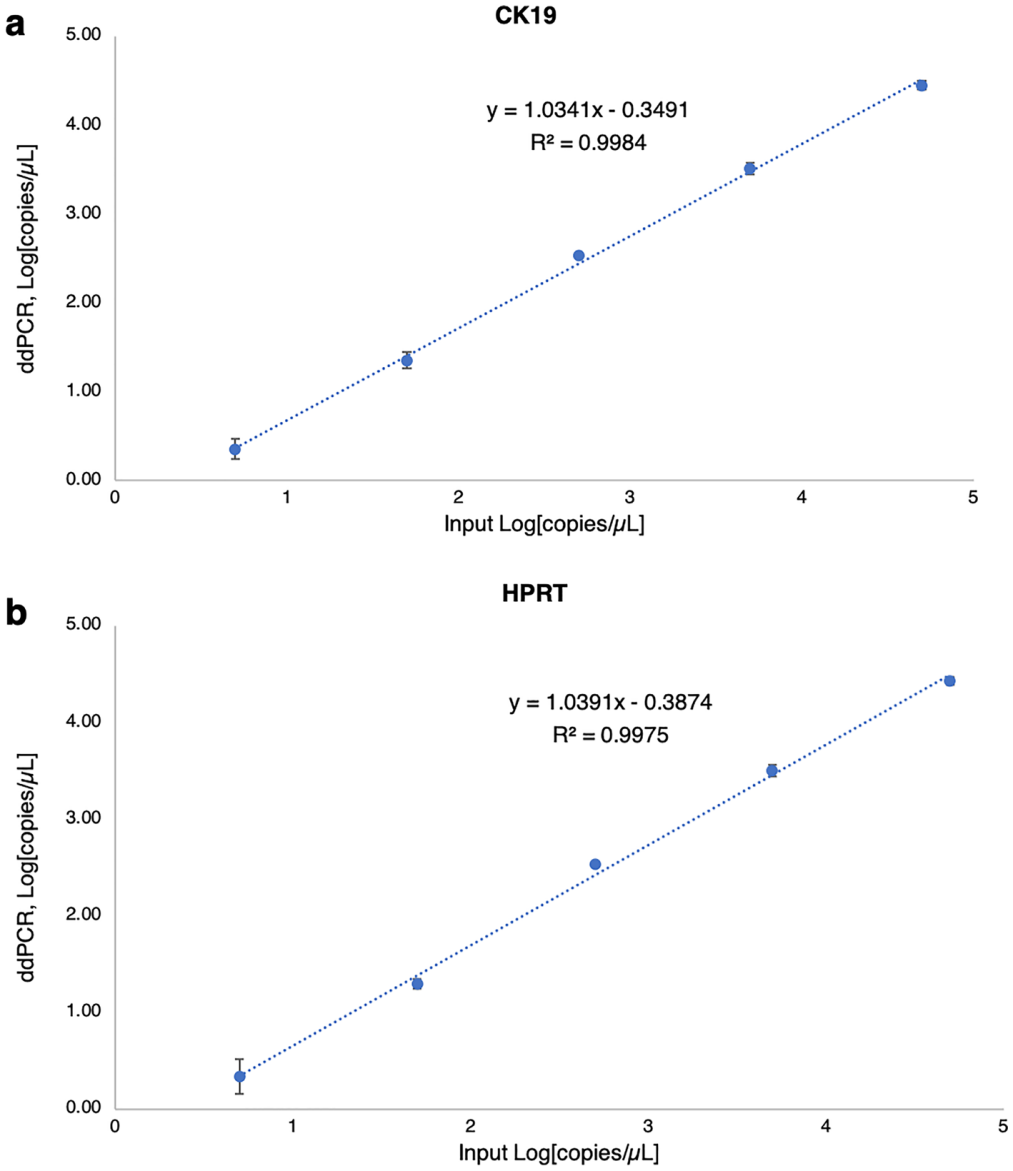
Measurement range for CK19 and HPRT detection by ddPCR. The linear range of CK19 (**a**) and HPRT (**b**) was specified using the synthetic plasmid DNA, in the range of 50,0000–5 copies/µL. DNA templates were plotted against input DNA using a spectrophotometer and against observed DNA using ddPCR. Error bars indicate standard deviations. The assays were performed in three replicates.

The measurement range of the duplex ddPCR method in CK19 and HPRT detection is shown in Fig. 4. It was found that the relationship between the input plasmid DNA measured by spectrophotometer and the observed plasmid concentration by ddPCR was linear. The correlation coefficients (R^2^) for CK19 and HPRT detection were 0.998 for both plots. At the lowest point of linear range, 5 copies/µL of plasmid DNA were used to determine the LOD and found that this concentration showed positive detection at 95% confidence (10 replicates). At this point, the ddPCR yielded 2.16 ± 1.27 and 2.44 ± 1.31 copies/µL for CK19 and HPRT, respectively (see Supplementary Fig. S15).

### CK19 and HPRT detection in canine peripheral blood and mammary tissue by ddPCR

**Figure 5:**
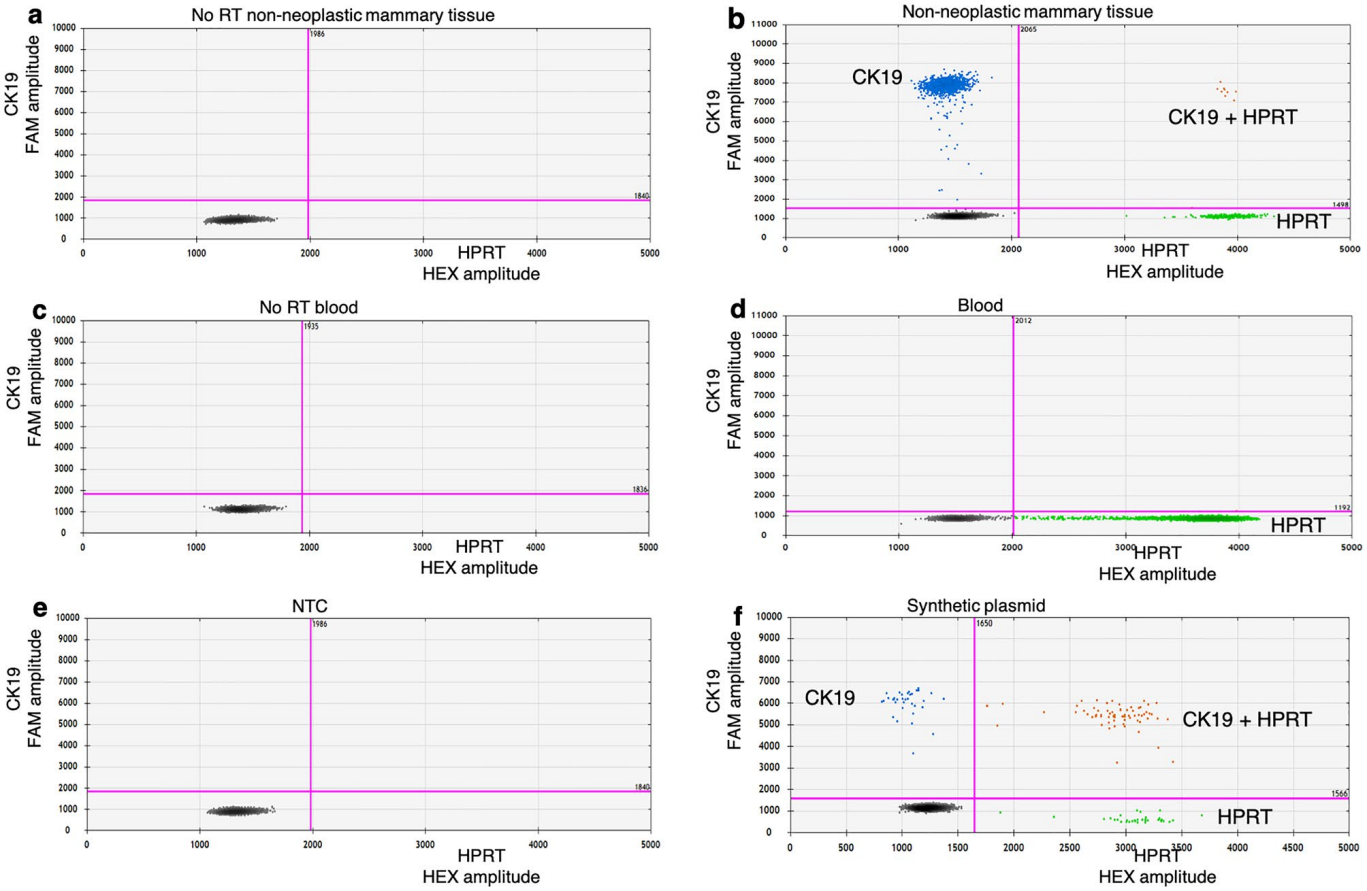
2D plots of duplex ddPCR results. The assays were tested with the templates; (**a**) no reverse transcriptase (no-RT) of non-neoplastic mammary tissue sample, (**b**) the cDNA derived from the non-neoplastic mammary tissue sample, (**c**) no-RT of blood sample, (**d**) the cDNA derived from blood sample, (**e**) no template control (NTC) and (**f**) synthetic plasmid DNA. Blue dots represent CK19, green dots are HPRT, orange dots contain both genes and black dots show when neither gene was detected.

**Figure 6:**
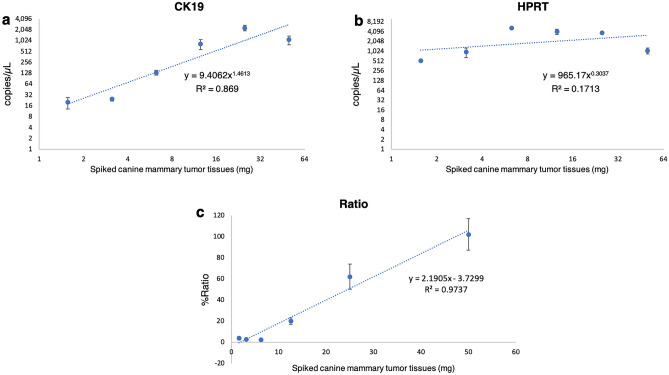
Correlations between copy numbers of CK19 (**a**), HPRT (**b**), copy number ratio (CK19/HPRT) (**c**) from duplex ddPCR assay and weight of spiked canine mammary tumor tissues (a twofold weight reduction from 50 to 1.56 mg) in the blood of healthy female dog. The copy numbers of CK19 and HPRT are shown in log_2_ scale. The error bar represents SD.

The optimized duplex ddPCR method for CK19 and HPRT detection was tested with RNA from peripheral blood and non-neoplastic mammary tissues. The ddPCR results showed that CK19 and HPRT genes were not found in no-RT controls from non-neoplastic mammary tissues and canine blood samples (Fig. 5a,c), nor in NTC (Fig. 5e), whereas CK19 and HPRT were both detected in cDNA from non-neoplastic mammary tissues (467 ± 79.3 copies/µL for CK19 and 79.5 ± 31.4 copies/µL for HPRT) (Fig. 5b) and in the synthetic plasmid DNA (239 ± 6.24 copies/µL for CK19 and 230 ± 6.56 copies/µL for HPRT) (Fig. 5f). However, in cDNA from canine blood samples, only HPRT (6005 ± 473.2 copies/µL) was observed by ddPCR (Fig. 5d). To verify the method, spiked samples (positive controls) were also investigated (see Supplementary Fig. S16). The results showed that CK19 and HPRT were detected in all spiked samples. And when the copy number ratio (copy number of CK19/copy number of HPRT) were calculated, the R^2^ = 0.9737 (Fig. 6).

## Discussion

In human medicine, detection of CK19 mRNA-positive cells in peripheral blood using PCR technique is beneficial to identify early-stage breast cancer patients with micrometastatic diseases and used for prognostications such as poor survival rate, disease free interval and overall survival time^7–11^. Detection of CK19 mRNA-positive cells has been reported in approximately 31–38%^7^ and 40%^10^ of early-stage breast cancer patients. However, there has been no study associated with this aspect in CMTs yet. We decided to utilize an advanced technology—namely, ddPCR—to investigate this gap, particularly the method validation. Although both qPCR and ddPCR can provide sensitive detection and precise quantification, qPCR itself depends on the relationship of the cycle threshold of a test sample to a standard curve. Moreover, distinct variation in assay PCR protocol and in materials used as calibration standards may be incomparable between different experiments, even when testing identical materials^15^. While ddPCR can provide an absolute quantification of target gene precisely without the standard curve. Furthermore, this advanced technology can find rare targets in complex backgrounds, detect small copy number variations (CNVs) and is highly tolerant to PCR inhibitors^26^. Many studies have demonstrated that ddPCR is a more robust approach for CNVs and gene detection for oncology and virology, compared to qPCR^14,27^. Therefore, in this study ddPCR was proposed as a promising technique for the study of CK19 and HPRT in canine gene expression tissue, and for detection of rare CTCs in canine blood.

In accordance with our samples of mammary tissues and whole blood samples, HPRT was selected as the reference gene in the present study. The advantage of the reference gene is not only due to its gene expression in normalization to ddPCR data, but it also acts as internal control gene that can verify the sample extraction and reverse transcription methods^28^. Previous studies have investigated the reference genes for dogs^29–32^. Their findings showed that HPRT was a good candidate for reference gene in dogs and HPRT is one of the most stable genes in mammary gland tissue and whole blood^29,30,32^, while GAPDH was less stable in canine mammary tissue^33^. In the results presented here, we found that canine blood and mammary tissues were rich in the HPRT gene, and it was concluded that HPRT gene can be used as reference gene in canine whole blood and mammary tissue, in line with previous studies^29,30,32^.

The T_a_ was optimized, and 55 °C was defined as the optimal temperature due to the highest concentrations of CK19 and HPRT genes being observed at this temperature. Both singleplex and duplex ddPCR were performed during optimization of T_a_. The results showed no differences in the concentrations of CK19 and HPRT genes between singleplex and duplex ddPCR assays. Hence, duplex ddPCR was selected for further investigation. The benefits of duplex ddPCR assays compared to singleplex are rapidity and cost-effectiveness^34^. Moreover, a duplex assay provides better precision in gene expression experiments because the target and normalizer genes are in the same well, thus, reducing technical errors as well as the amount of sample required.

The LOD of the duplex assays was assessed to define the lowest concentration which can be discriminated with a level of confidence of 95%^25^. The optimal assay was tested with RNA from peripheral blood and non-neoplastic mammary tissues. In canine mammary tissue, the expression of CK19 and HPRT was detected, while in the canine blood sample only HPRT was determined in both methods (qPCR and ddPCR). These results confirmed that the assays were verified. CK19 mRNA was not found in the blood of a healthy dog, this may be due to there being no CTCs containing the CK19 gene in the blood circulation, as reported in healthy humans^7–11^. However, in clinical cases, false negative would be happened if CTCs were negative for CK19. Few studies reported that CK19 mRNA-positive CTCs may be found in human patients with hepatocellular carcinoma^35^ and benign colon diseases^36^ and these cells might be a source of false-positive findings. Although, to our knowledge, there are no reports showing CK19 mRNA-positive CTCs in dogs suffering from hepatocellular carcinoma and/or colon diseases, any potential factors resulting in false-positive findings must be ruled out and further studies with this regard should be investigated. Our result was considered trustworthy due to detection of HPRT gene expression in the blood samples. In addition, this developed method was verified using spiked canine mammary tumor tissues in the blood of healthy female dog as positive control. In our study, RNA samples were treated with DNase I, and no-RT controls were performed to assess the amount of DNA contamination, and the results showed that no CK19 and HPRT were detected. In addition, NTC was conducted and served as a general control for extraneous nucleic acid contamination. Consequently, all steps were proven and stable for detection of CK19 mRNA in clinical samples.

## Conclusions

In conclusion, this work presented newly designed primers and probes for CK19 and HPRT in canine peripheral blood and mammary tissue. The assay was tested with RNA from peripheral blood, non-neoplastic mammary tissues and spiked samples. The outcomes supported that all materials and methods used in this study were valid, and can be applied for CTCs via CK19 mRNA detection in canine blood of CMT patients, and for gene expression in canine mammary neoplastic tissues using ddPCR. In veterinary oncology, further investigation of CK19 mRNA detection in clinical cases could be beneficial for CMT prognostication.

## Supplementary Information

Supplementary Information.
