# Supplementary material for: Validation of droplet digital PCR for cytokeratin 19 mRNA detection in canine peripheral blood and mammary gland
Source: Sci Rep. 2022 Aug 10;12:13623. doi: 10.1038/s41598-022-17493-5 (PMC9365843; doi:10.1038/s41598-022-17493-5)
Supplement: Supplementary file 1 — Supplementary Information. [file 41598_2022_17493_MOESM1_ESM.pdf]

Validation of droplet digital PCR for cytokeratin 19 mRNA detection in canine peripheral blood and mammary gland

Potsawat Tanvetthayanont[1], Teerapong Yata[2], Jiranun Boonnil[3], Sasithon Temisak[3, *], Suppawiwat Ponglowhapan[1, *]

[1]Department of Obstetric Gynaecology and Reproduction, Faculty of Veterinary Science, Chulalongkorn University, Bangkok, Thailand, 10330. Email: tanvet.pt@gmail.com

[2]Unit of Biochemistry, Department of Physiology, Faculty of Veterinary Science, Chulalongkorn University, Bangkok, Thailand, 10330. Email: teerapong.y@chula.ac.th

[3]National Institute of Metrology (NIMT), Pathumthani, Thailand, 12120. Email: sasithont@nimt.or.th

*Corresponding authors

: Suppawiwat Ponglowhapan. E-mail**:** sponglowhapan@gmail.com

: Sasithon Temisak. E-mail: sasithont@nimt.or.th

Supplementary legends

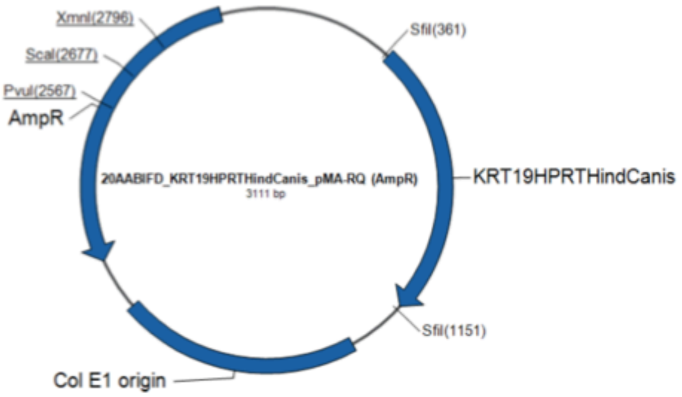

>Template

AAGCTTCTGGTACCAGAGGCAGGGGCCCGGGCCCGCCCGCGAGTACAGTGAGTCCTAAAAGACCATCGAGGACCTGCGGGACAAGATTCTTGGTGCCACCATTG
AGAACTCCAAGATTGTCCTGCAGATTGACAATGCCCGTCTGGCTGCGGATGACTTCCGAACCAAGTTTGAGACGGAGCAGGCCCTGCGCATGAGTGTGGAGGCTG
ACATCAATGGCCTGCGCCGGGTGCTGGATGAGCTGACCTTGGCCAGAGCTGATCTGGAGTTGCAGATCGAAGGCCTAAGCTTCATGGACTAATTATGGACAGGAC
TGAGCGGCTTGCTCGAGATGTGATGAAGGAAATGGGAGGCCATCACATCGTAGCCCTCTGTGTGCTCAAGGGAGGCTATAAATTCTTTGCTGACCTGCTGGATTA
TATCAAAGCACTGAACAGAAATAGTGATGGATCCATTCCTATGACTGTAGATTTCATCAGACTGAAGAGCTACTGTAATGACCAGTCAACAGGGGACATAAAAGTA
ATTGGTGGAGATGATCTCTCAACTTTAACTGGAAAGAATGTCTTGATTGTTGAGGATATAATTGACACTGGGAAAACAATGCAGACTTTGCTTTCCTTGGTCAAGG
AGCATAATCCAAAGATGGTCAAGGTTGCAAGCTTGCTGGTGAAAAGGACCCCTCGAAGTGTTGGCTATAAACCTGACTTTGTTGGATTTGAAATTCCAGACAAGT
TTGTTGTAGGATATGCCCTTGACTATAAGAATTC

Green; HindIII site, Red; CK19 sequences, Blue; HPRT sequences, Purple; EcoRI site (plasmid size; 3111 bp and gene size; 770 bp).

Figure S1. Plasmid map and sequences. Red indicate the CK19 fragment located on 339 to 617 of CK19 mRNA gene (NM_001253742.1). Blue indicate the HPRT fragment located on 115 to 587 of HPRT mRNA gene (AY283372.1).

ATGACTTCCTACAGCTACCGCCACTCGTCCGCCACCTCGTCCTTCGGGGGCCTGGGCGGCGGCTCCCTGCGCCT
CGGGCCGGGCGGTGCCTTCCGCGCGCCCAGCATCCACGGGGGCTCGGGCGGCCGCGGCGTGTCTGTGTCCTCCG
CCCGCTTCGTGTCCTCGTCCTCCGGGGGCTACGGCGGCGGCTTCACGAGCGGCCTGGGCCGGTCCGATGGGCTG
CTGGCGGGCAATGAGAAGCTCACCATGCAGAACCTCAACGACCGCCTGGCCTCCTACCTGGACAAGGTGCGCGC
CCTGGAGGAGGCCAACGGCGACCTGGAGGTGAAGATCCGCGACTGGTACCAGAGGCAGGGGCCCGGGCCCGCCC
GCGAGTACAGTGAGTCCTAAAAGACCATCGAGGACCTGCGGGACAAGATTCTTGGTGCCACCATTGAGAACTCC
AAGATTGTCCTGCAGATTGACAATGCCCGTCTGGCTGCGGATGACTTCCGAACCAAGTTTGAGACGGAGCAGGC
CCTGCGCATGAGTGTGGAGGCTGACATCAATGGCCTGCGCCGGGTGCTGGATGAGCTGACCTTGGCCAGAGCTG
ATCTGGAGTTGCAGATCGAAGGCCTGAAGGAGGAGCTGGCCTACCTGAAGAAGAACCACGAGGAGGAAATCAGT
GCCCTGAGGGGCCAGGTGGGTGGCCAGGTCAGTGTGGAGGTGGATTCCGCTCCTGGCATTGACCTTGCCAAAAT
CCTGAGTGACATGAGAAGCCAATATGAAGTCATGGCTGAGAAGAACCGGAAGGATGCTGAAGCCTGGTTCACCA
GCCGGACTGAGGAGCTGAGTCGGGAGGTGGCCGGCCACACAGAGCAGCTGCAGATAAGCAAGACGGAGGTCACT
GACCTGCGGCGCACCCTCCAGGGTCTGGAGATCGAGCTGCAGTCTCAGCTAAGCATGAAAGCTGCCCTGGAAGG
CACACTGGCGGAAACAGAGGCCCGCTTTGGAGCCCAGCTGGCCCAGATCCAGGCTCTGATCAGCAGCATGGAAG
CCCAGCTGAGCGATGTGCGTGCGGACACTGAACGGCAGAACCAGGAGTACCAGCAGCTCATGGACATCAAGTCA
CGGCTGGAGCAGGAGATTGCCACCTACCGCAGCTTGCTGGAGGGCCAGGACGCCCACTACAACAACCTGCCCAC
CCCCAAGGCTCTCTGAGTCCAGCAGCCTCCTGGGCTCCCTGCTCTCCTGCGGATGGGGTGCCCCTGGGTAGGGC
CATGGGAGGGGAGGGACCCTACCCCTGGCTCTTTCCCTGACCTGCCAATAAAGCTTTATGGCTCCAGGAGGGAT
GTTGGGTCTGTTTTCTCAGTCCATAAAGATGGGCCAGAACTTGGTCCTGTTTAGGATTTATTTGGATCAGAATA
CACCCTGTATTCCCAGGAGAGGGAGGGAGGGTGCTCACCAACTCCTACTGTTATGGTAATTCCTGTTTGCAGAA
CTCTTCTTCATTTATCTAACCTATTCCTTAAAACAAGCTTGTGAAATAAACATTGCTCCCCTCTTTAAAAAAAA

Figure S2. The location of forward and reverse primers (yellow), and probe (green) for CK19 on *Canis lupus familiaris* keratin 19 mRNA (NM_001253742.1). Reference genome: CanFam3.1 (GCF_000002285.3), Location NC_006591.3 (21240778..21245039).

ATGGCGACCCGCAGCCTTGGCGTCGTGATTAGTGATGATGAACCAGGTTATGACCTAGATTTATTTTGTATACC
TCATCATTACGCTGAGGATTTGGAAAAAGTGTTTATTCCTCATGGACTAATTATGGACAGGACTGAGCGGCTTG
CTCGAGATGTGATGAAGGAAATGGGAGGCCATCACATCGTAGCCCTCTGTGTGCTCAAGGGAGGCTATAAATTC
TTTGCTGACCTGCTGGATTATATCAAAGCACTGAACAGAAATAGTGATGGATCCATTCCTATGACTGTAGATTT
CATCAGACTGAAGAGCTACTGTAATGACCAGTCAACAGGGGACATAAAAGTAATTGGTGGAGATGATCTCTCAA
CTTTAACTGGAAAGAATGTCTTGATTGTTGAGGATATAATTGACACTGGGAAAACAATGCAGACTTTGCTTTCC
TTGGTCAAGGAGCATAATCCAAAGATGGTCAAGGTTGCAAGCTTGCTGGTGAAAAGGACCCCTCGAAGTGTTGG
CTATAAACCTGACTTTGTTGGATTTGAAATTCCAGACAAGTTTGTTGTAGGATATGCCCTTGACTATAATGAAT
ACTTCAGGGATTTGAATCATGTTTGTGTCATTAGTGAAACTGGAAAAGCAAAATACAAAGCCTAAGATGAGAGT
TCAAGTTGAGTTTGGAAGCATCTGGAGTCCCATTGAATTCATATCATCGGTAGAATGTTCTAGTTCTGTGGCCA
TCTGCTTAGTAAAGCTTTTTGCATG

Figure S3. The location of forward and reverse primers (yellow), and probe (green) for HPRT on *Canis familiaris* hypoxanthine phosphoribosyl transferase (HPRT) mRNA (AY283372.1). Reference genome: CanFam3.1 (GCF_000002285.3) Location NC_006621.3 (105115732..105153702).

Figure S4. Shows mRNA sequences of CK19 between human (*Homo sapiens* keratin 19 mRNA; NM_002276.5) and dog (*Canis lupus familiaris* keratin 19 mRNA; NM_001253742.1). Asterisk represents the match base. The location of forward and reverse primers (yellow), and probe (green) for CK19 on *Canis lupus familiaris* keratin 19, mRNA (NM_001253742.1).

```
NM_002276.5          GCTCCTCCCGCGAATCGCAGCTTCTGAGACCAGGGTTGCTCCGTCCGTGCTCCGCCTCGC
NM_001253742.1       ------------------------------------------------------------

NM_002276.5          CATGACTTCCTACAGCTATCGCCAGTCGTCGGCCACGTCGTCCTTCGGAGGCCTGGGCGG
NM_001253742.1       -ATGACTTCCTACAGCTACCGCCACTCGTCCGCCACCTCGTCCTTCGGGGGCCTGGGCGG
                      ***************** ***** ***** ***** ***********.***********
NM_002276.5          CGGCTCCGTGCGTTTTGGGCCGGGGGTCGCCTTTCGCGCGCCCAGCATTCACGGGGGCTC
NM_001253742.1       CGGCTCCCTGCGCCTCGGGCCGGGCGGTGCCTTCCGCGCGCCCAGCATCCACGGGGGCTC
                     ******* ****  * ******** *  ***** ************** ***********
NM_002276.5          CGGCGGCCGCGGCGTATCCGTGTCCTCCGCCCGCTTTGTGTCCTCGTCCTCCTCGGGGGC
NM_001253742.1       GGGCGGCCGCGGCGTGTCTGTGTCCTCCGCCCGCTTCGTGTCCTCGTCCTCCGGGGG---
                      **************.** ***************** ***************  ***
NM_002276.5          CTACGGCGGCGGCTACGGCGGCGTCCTGACCGCGTCCGACGGGCTGCTGGCGGGCAACGA
NM_001253742.1       CTACGGCGGCGGCTTCACGAGCGGCCTGGGCCGGTCCGATGGGCTGCTGGCGGGCAATGA
                     **************:*.  .*** ****. *  ****** ***************** **
NM_002276.5          GAAGCTAACCATGCAGAACCTCAACGACCGCCTGGCCTCCTACCTGGACAAGGTGCGCGC
NM_001253742.1       GAAGCTCACCATGCAGAACCTCAACGACCGCCTGGCCTCCTACCTGGACAAGGTGCGCGC
                     ******.*****************************************************
NM_002276.5          CCTGGAGGCGGCCAACGGCGAGCTAGAGGTGAAGATCCGCGACTGGTACCAGAAGCAGGG
NM_001253742.1       CCTGGAGGAGGCCAACGGCGACCTGGAGGTGAAGATCCGCGACTGGTACCAGAGGCAGGG
                     ********.************ **.****************************.******
NM_002276.5          GCCTGGGCCCTCCCGCGACTACAGCCACTACTACACGACCATCCAGGACCTGCGGGACAA
NM_001253742.1       GCCCGGGCCCGCCCGCGACTACAGCCACTACTTCAAGACCATCGAGGACCTGCGGGACAA
                     *** ****** *********************:**.******* ****************
NM_002276.5          GATTCTTGGTGCCACCATTGAGAACTCCAGGATTGTCCTGCAGATCGACAATGCCCGTCT
NM_001253742.1       GATTCTTGGTGCCACCATTGAGAACTCCAAGATTGTCCTGCAGATTGACAATGCCCGTCT
                     *****************************.*************** **************
NM_002276.5          GGCTGCAGATGACTTCCGAACCAAGTTTGAGACGGAACAGGCTCTGCGCATGAGCGTGGA
NM_001253742.1       GGCTGCGGATGACTTCCGAACCAAGTTTGAGACGGAGCAGGCCCTGCGCATGAGTGTGGA
                     ******.*****************************.***** *********** *****
NM_002276.5          GGCCGACATCAACGGCCTGCGCAGGGTGCTGGATGAGCTGACCCTGGCCAGGACCGACCT
NM_001253742.1       GGCTGACATCAATGGCCTGCGCCGGGTGCTGGATGAGCTGACCTTGGCCAGAGCTGATCT
                     *** ******** *********.******************** *******..* ** **
NM_002276.5          GGAGATGCAGATCGAAGGCCTGAAGGAAGAGCTGGCCTACCTGAAGAAGAACCATGAGGA
NM_001253742.1       GGAGTTGCAGATCGAAGGCCTGAAGGAGGAGCTGGCCTACCTGAAGAAGAACCACGAGGA
                     ****:**********************.************************** *****
NM_002276.5          GGAAATCAGTACGCTGAGGGGCCAAGTGGGAGGCCAGGTCAGTGTGGAGGTGGATTCCGC
NM_001253742.1       GGAAATCAGTGCCCTGAGGGGCCAGGTGGGTGGCCAGGTCAGTGTGGAGGTGGATTCCGC
                     **********.* ***********.*****:*****************************
NM_002276.5          TCCGGGCACCGATCTCGCCAAGATCCTGAGTGACATGCGAAGCCAATATGAGGTCATGGC
NM_001253742.1       TCCTGGCATTGACCTTGCCAAAATCCTGAGTGACATGAGAAGCCAATATGAAGTCATGGC
                     *** ****  ** ** *****.***************.*************.********
NM_002276.5          CGAGCAGAACCGGAAGGATGCTGAAGCCTGGTTCACCAGCCGGACTGAAGAATTGAACCG
NM_001253742.1       TGAGAAGAACCGGAAGGATGCTGAAGCCTGGTTCACCAGCCGGACTGAGGAGCTGAGTCG
                      ***.*******************************************.**. ***. **
NM_002276.5          GGAGGTCGCTGGCCACACGGAGCAGCTCCAGATGAGCAGGTCCGAGGTTACTGACCTGCG
NM_001253742.1       GGAGGTGGCCGGCCACACAGAGCAGCTGCAGATAAGCAAGACGGAGGTCACTGACCTGCG
                     ****** ** ********.******** *****.****.*:* ***** ***********
NM_002276.5          GCGCACCCTTCAGGGTCTTGAGATTGAGCTGCAGTCACAGCTGAGCATGAAAGCTGCCTT
NM_001253742.1       GCGCACCCTCCAGGGTCTGGAGATCGAGCTGCAGTCTCAGCTAAGCATGAAAGCTGCCCT
                     ********* ******** ***** ***********:*****.*************** *
NM_002276.5          GGAAGACACACTGGCAGAAACGGAGGCGCGCTTTGGAGCCCAGCTGGCGCATATCCAGGC
```

```
NM_001253742.1      GGAAGGCACACTGGCGGAAACAGAGGCCCGCTTTGGAGCCCAGCTGGCCCAGATCCAGGC
                    *****.*********.*****.***** ******************** ** ********

NM_002276.5         GCTGATCAGCGGTATTGAAGCCCAGCTGGGCGATGTGCGAGCTGATAGTGAGCGGCAGAA
NM_001253742.1      TCTGATCAGCAGCATGGAAGCCCAGCTGAGCGATGTGCGTGCGGACACTGAACGGCAGAA
                     *********.* ** ************.**********:** ** * ***.********

NM_002276.5         TCAGGAGTACCAGCGGCTCATGGACATCAAGTCGCGGCTGGAGCAGGAGATTGCCACCTA
NM_001253742.1      CCAGGAGTACCAGCAGCTCATGGACATCAAGTCACGGCTGGAGCAGGAGATTGCCACCTA
                     *************.******************.**************************

NM_002276.5         CCGCAGCCTGCTCGAGGGACAGGAAGATCACTACAACAATTTGTCTGCCTCCAAGGTCCT
NM_001253742.1      CCGCAGCTTGCTGGAGGGCCAGGACGCCCACTACAACAACCTGCCCACCCCCAAGGCTCT
                    ******* **** *****.*****.*. ***********  ** * .** ******  **

NM_002276.5         CTGAG-GCAGCAGGCTCTGGGGCTTCTG-CTGTCCTTTGGAGGGTGT-CTTCTGGGTAGA
NM_001253742.1      CTGAGTCCAGCAGCCTCCTGGGCTCCCTGCTCTCCTGCGGATGGGGTGCCCCTGGGTAGG
                    *****  ****** ***  ***** *   ** ****  *** ** ** *  ********.

NM_002276.5         GGGATGGGAAGGAAGGGACCCTTACCCCCGGCTCTTCTCCTGACCTGCCAATAAAAATTT
NM_001253742.1      GCCATGGGAGGGGAGGGACCCT-ACCCCTGGCTCTTTCCCTGACCTGCCAATAAAGCTTT
                    *  ******.**.********* ***** *******  *****************..***

NM_002276.5         ATGGTCCAAGGGA-----------------------------------------------
NM_001253742.1      ATGGCTCCAGGAGGGATGTTGGGTCTGTTTTCTCAGTCCATAAAGATGGGCCAGAACTTG
                    ****  *.***..

NM_002276.5         ------------------------------------------------------------
NM_001253742.1      GTCCTGTTTAGGATTTATTTGGATCAGAATACACCCTGTATTCCCAGGAGAGGGAGGGAG

NM_002276.5         ------------------------------------------------------------
NM_001253742.1      GGTGCTCACCAACTCCTACTGTTATGGTAATTCCTGTTTGCAGAACTCTTCTTCATTTAT

NM_002276.5         -----------------------------------------------------------
NM_001253742.1      CTAACCTATTCCTTAAAACAAGCTTGTGAAATAAACATTGCTCCCCTCTTTAAAAAAAA
```

Figure S5. Shows mRNA sequences of HPRT between human (*Homo sapiens* hypoxanthine phosphoribosyl transferase mRNA; NM_000194.3) and dog (*Canis familiaris* hypoxanthine phosphoribosyl transferase mRNA; AY283372.1). Asterisk represents the match base. The location of forward and reverse primers (yellow), and probe (green) for HPRT on *Canis familiaris* hypoxanthine phosphoribosyl transferase, mRNA (AY283372.1).

```
NM_000194.3      AGCTTCAGGCGGCTGCGACGAGCCCTCAGGCGAACCTCTCGGCTTTCCCGCGCGGCGCCG
AY283372.1       ------------------------------------------------------------

NM_000194.3      CCTCTTGCTGCGCCTCCGCCTCCTCCTCTGCTCCGCCACCGGCTTCCTCCTCCTGAGCAG
AY283372.1       ------------------------------------------------------------

NM_000194.3      TCAGCCCGCGCGCCGGCCGGCTCCGTTATGGCGACCCGCAGCCCTGGCGTCGTGATTAGT
AY283372.1       ---------------------------ATGGCGACCCGCAGCCTTGGCGTCGTGATTAGT
                                            **************** ****************
NM_000194.3      GATGATGAACCAGGTTATGACCTTGATTTATTTTGCATACCTAATCATTATGCTGAGGAT
AY283372.1       GATGATGAACCAGGTTATGACCTAGATTTATTTTGTATACCTCATCATTACGCTGAGGAT
                 ***********************:*********** ******.******* *********
NM_000194.3      TTGGAAAGGGTGTTTATTCCTCATGGACTAATTATGGACAGGACTGAACGTCTTGCTCGA
AY283372.1       TTGGAAAAAGTGTTTATTCCTCATGGACTAATTATGGACAGGACTGAGCGGCTTGCTCGA
                 *******..**************************************.** *********
NM_000194.3      GATGTGATGAAGGAGATGGGAGGCCATCACATTGTAGCCCTCTGTGTGCTCAAGGGGGGC
AY283372.1       GATGTGATGAAGGAAATGGGAGGCCATCACATCGTAGCCCTCTGTGTGCTCAAGGGAGGC
                 **************.***************** ***********************.***
NM_000194.3      TATAAATTCTTTGCTGACCTGCTGGATTACATCAAAGCACTGAATAGAAATAGTGATAGA
AY283372.1       TATAAATTCTTTGCTGACCTGCTGGATTATATCAAAGCACTGAACAGAAATAGTGATGGA
                 ***************************** ************** ************.**
NM_000194.3      TCCATTCCTATGACTGTAGATTTTATCAGACTGAAGAGCTATTGTAATGACCAGTCAACA
AY283372.1       TCCATTCCTATGACTGTAGATTTCATCAGACTGAAGAGCTACTGTAATGACCAGTCAACA
                 *********************** ***************** ******************
NM_000194.3      GGGGACATAAAAGTAATTGGTGGAGATGATCTCTCAACTTTAACTGGAAAGAATGTCTTG
AY283372.1       GGGGACATAAAAGTAATTGGTGGAGATGATCTCTCAACTTTAACTGGAAAGAATGTCTTG
                 ************************************************************
NM_000194.3      ATTGTGGAAGATATAATTGACACTGGCAAAACAATGCAGACTTTGCTTTCCTTGGTCAGG
AY283372.1       ATTGTTGAGGATATAATTGACACTGGGAAAACAATGCAGACTTTGCTTTCCTTGGTCAAG
                 ***** **.***************** *******************************.*
NM_000194.3      CAGTATAATCCAAAGATGGTCAAGGTCGCAAGCTTGCTGGTGAAAAGGACCCCACGAAGT
AY283372.1       GAGCATAATCCAAAGATGGTCAAGGTTGCAAGCTTGCTGGTGAAAAGGACCCCTCGAAGT
                  ** ********************** **************************:******
NM_000194.3      GTTGGATATAAGCCAGACTTTGTTGGATTTGAAATTCCAGACAAGTTTGTTGTAGGATAT
AY283372.1       GTTGGCTATAAACCTGACTTTGTTGGATTTGAAATTCCAGACAAGTTTGTTGTAGGATAT
                 *****.*****.**:*********************************************
NM_000194.3      GCCCTTGACTATAATGAATACTTCAGGGATTTGAATCATGTTTGTGTCATTAGTGAAACT
AY283372.1       GCCCTTGACTATAATGAATACTTCAGGGATTTGAATCATGTTTGTGTCATTAGTGAAACT
                 ************************************************************
NM_000194.3      GGAAAAGCAAAATACAAAGCCTAAGATGAGAGTTCAAGTTGAGTTTGGAAACATCTGGAG
AY283372.1       GGAAAAGCAAAATACAAAGCCTAAGATGAGAGTTCAAGTTGAGTTTGGAAGCATCTGGAG
                 **************************************************.*********
NM_000194.3      TCCTATTGACATCGCCAGTAAAATTATCAATGTTCTAGTTCTGTGGCCATCTGCTTAGTA
AY283372.1       TCCCATTGAATTCATATCATCGGTAG--AATGTTCTAGTTCTGTGGCCATCTGCTTAGTA
                 *** *****.:**. .: ::...*:.  ********************************
NM_000194.3      GAGCTTTTTGCATGTATCTTCTAAGAATTTTATCTGTTTTGTACTTTAGAAATGTCAGTT
AY283372.1       AAGCTTTTTGCATG----------------------------------------------
                 .*************
NM_000194.3      GCTGCATTCCTAAACTGTTTATTTGCACTATGAGCCTATAGACTATCAGTTCCCTTTGGG
AY283372.1       ------------------------------------------------------------

NM_000194.3      CGGATTGTTGTTTAACTTGTAAATGAAAAAATTCTCTTAAACCACAGCACTATTGAGTGA
AY283372.1       ------------------------------------------------------------
```

```
NM_000194.3      AACATTGAACTCATATCTGTAAGAAATAAAGAGAAGATATATTAGTTTTTTAATTGGTAT
AY283372.1       ------------------------------------------------------------

NM_000194.3      TTTAATTTTTATATATGCAGGAAAGAATAGAAGTGATTGAATATTGTTAATTATACCACC
AY283372.1       ------------------------------------------------------------

NM_000194.3      GTGTGTTAGAAAAGTAAGAAGCAGTCAATTTTCACATCAAAGACAGCATCTAAGAAGTTT
AY283372.1       ------------------------------------------------------------


NM_000194.3      TGTTCTGTCCTGGAATTATTTTAGTAGTGTTTCAGTAATGTTGACTGTATTTTCCAACTT
AY283372.1       ------------------------------------------------------------

NM_000194.3      GTTCAAATTATTACCAGTGAATCTTTGTCAGCAGTTCCCTTTTAAATGCAAATCAATAAA
AY283372.1       ------------------------------------------------------------

NM_000194.3      TTCCCAAAAATTTAA
AY283372.1       ---------------
```

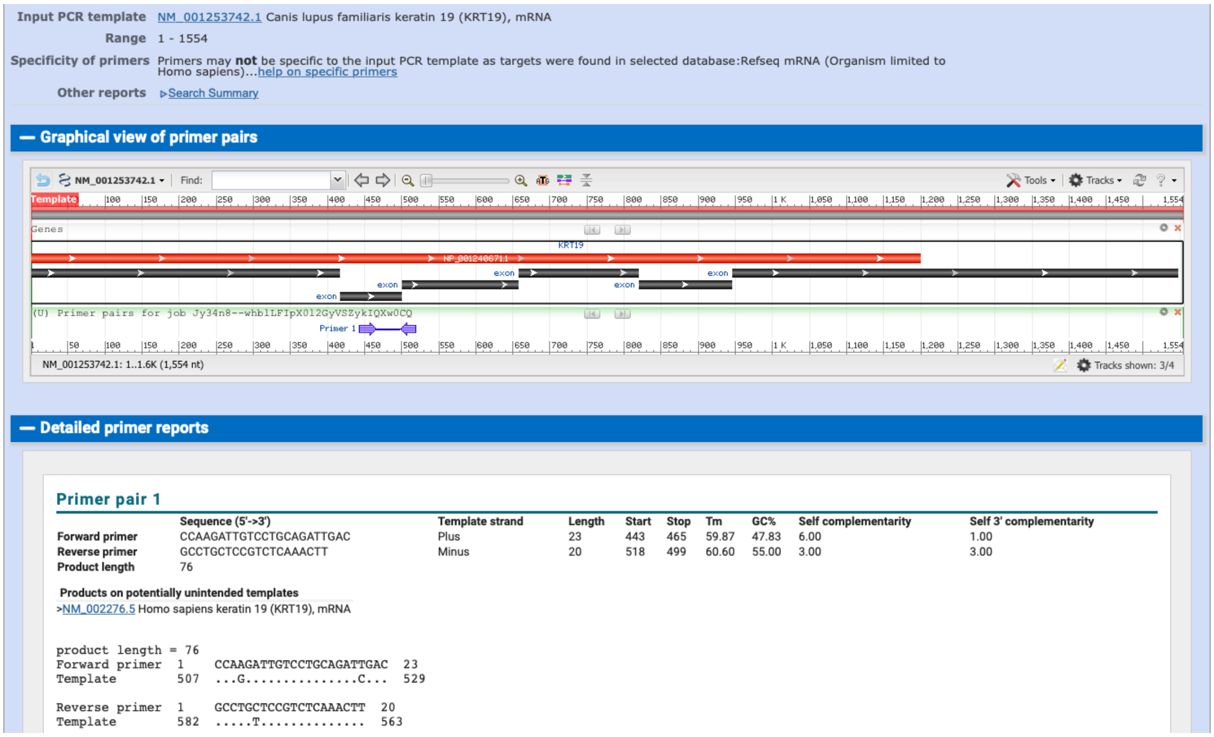

Figure S6. The specificity and binding location of CK19 primers to the *Canis lupus familiaris* keratin 19 mRNA (NM_001253742.1).

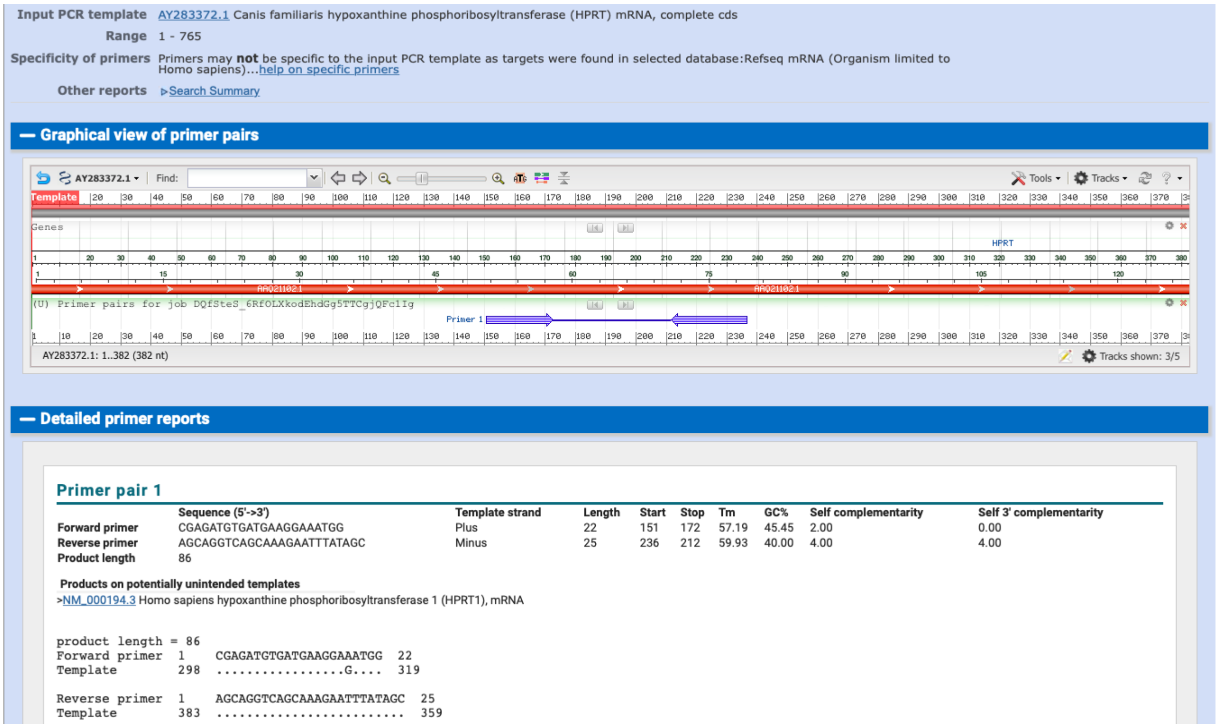

Figure S7. The specificity and binding location of HPRT primers to the *Canis familiaris* hypoxanthine phosphoribosyltransferase mRNA (AY283372.1).

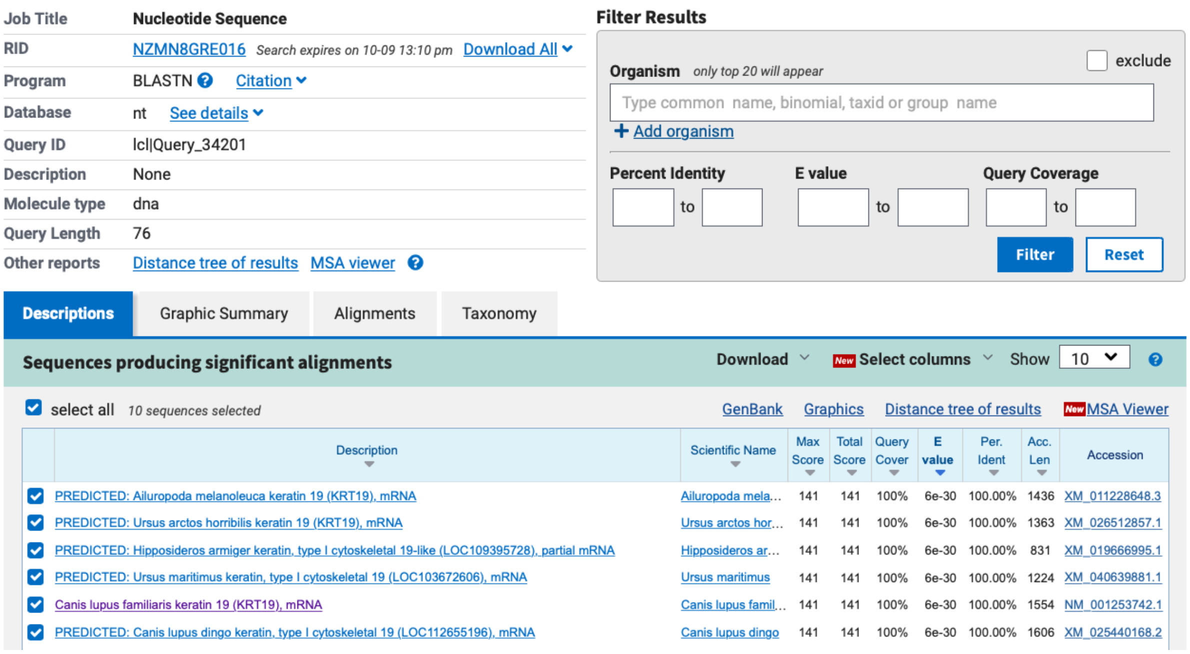

Figure S8. The specificity of CK19 probe design obtaining 100% match rate to the *Canis lupus familiaris* keratin 19 mRNA (NM_001253742.1).

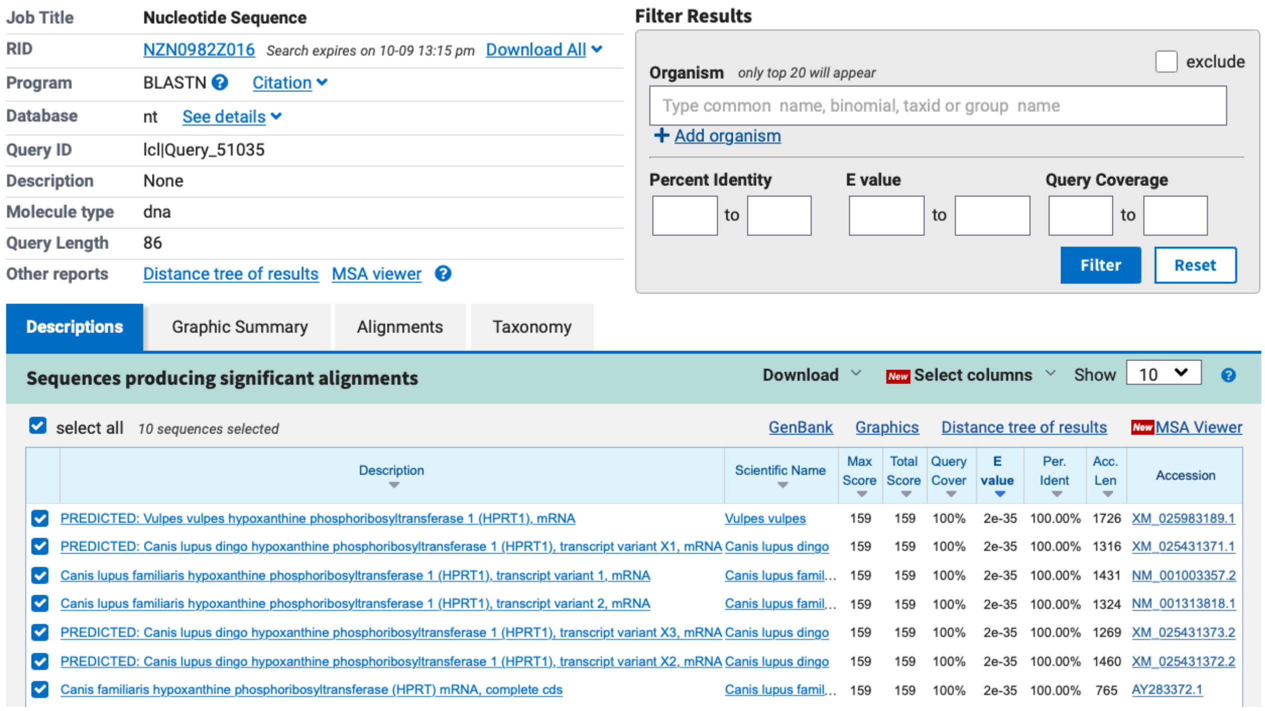

Figure S9. The specificity of HPRT probe design obtaining 100% match rate to the *Canis familiaris* hypoxanthine phosphoribosyltransferase mRNA (AY283372.1).

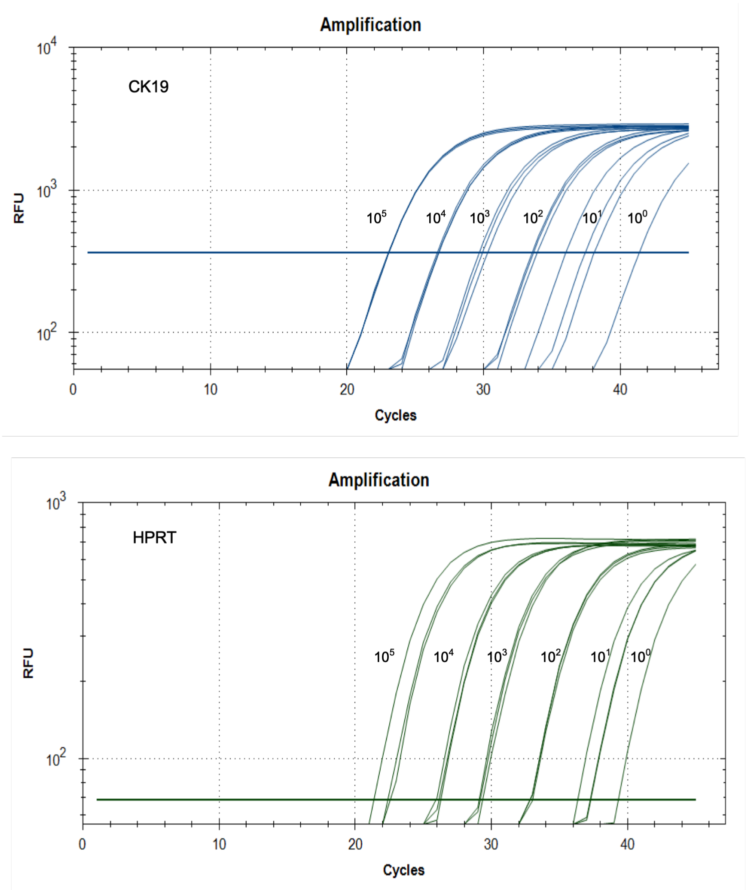

Figure S10. Amplification curves of CK19 and HPRT detection, using 10^0 to 10^5 copies of plasmid DNA templates.

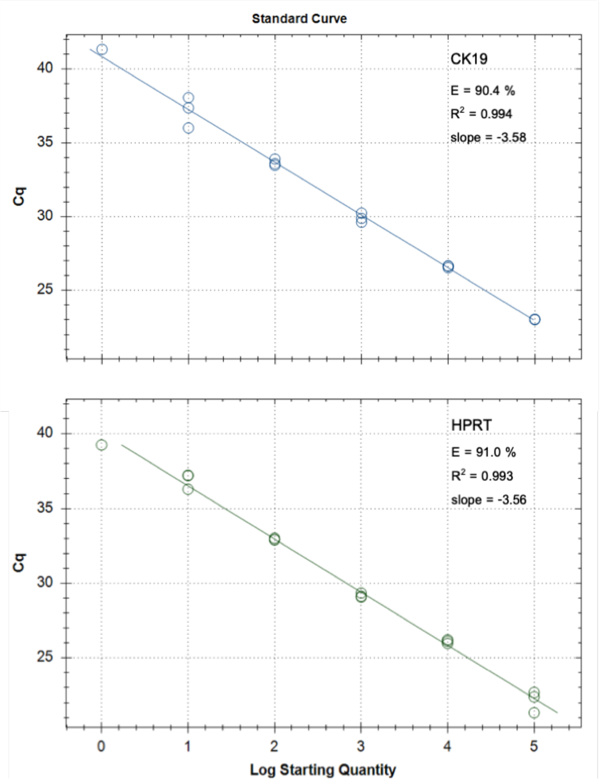

Figure S11. The standard curve shows the efficacies of qPCR for CK19 and HPRT detections with 90.4 % (R^2 = 0.994, slope = -3.58) and 91.0 % (R^2 = 0.993, slope = -3.56), respectively, using 10^0 to 10^5 copies of plasmid DNA templates.

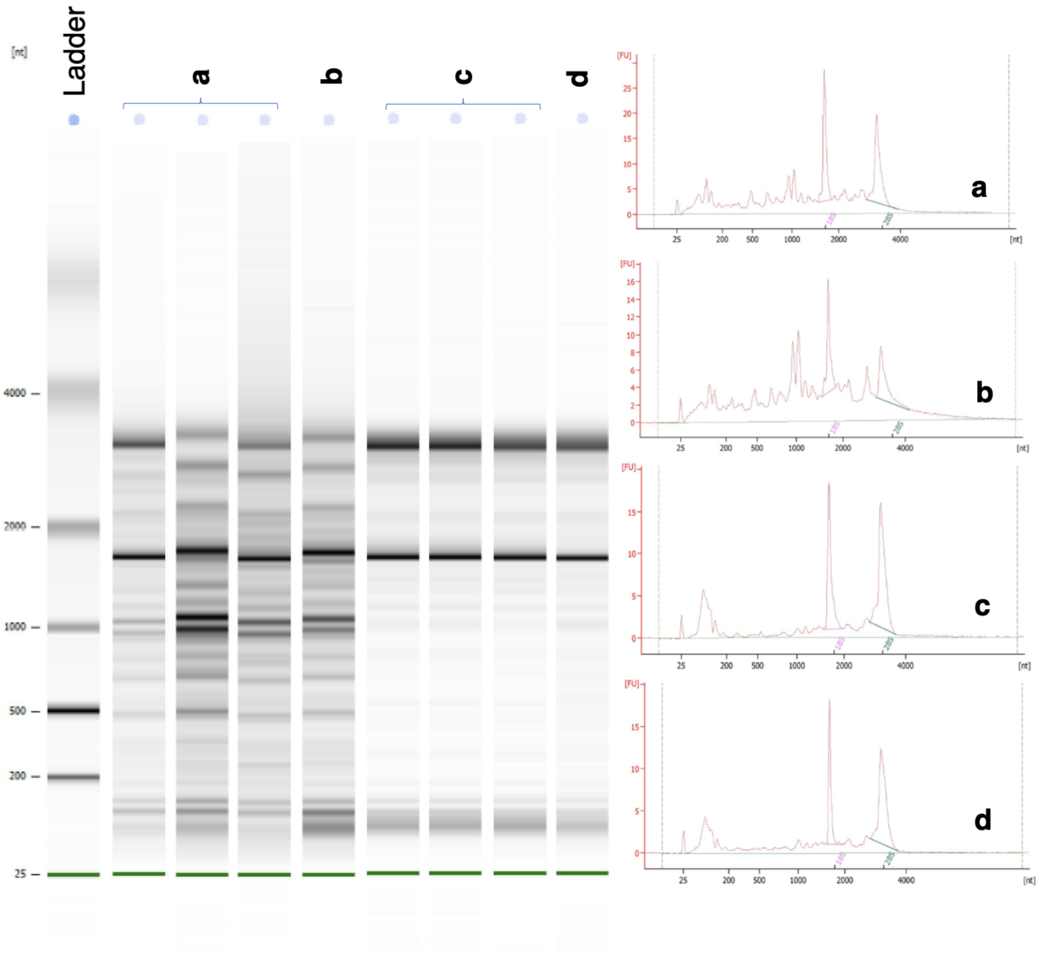

Figure S12. Show the electropherograms of mRNAs of non-neoplastic canine mammary tissues (RIN: 6.0 - 6.4) (a), canine mammary tumor tissue (RIN: 6.2) (b), peripheral blood (RIN: 8.6 – 8.8) (c) and spiked sample (RIN: 8.3) (d).

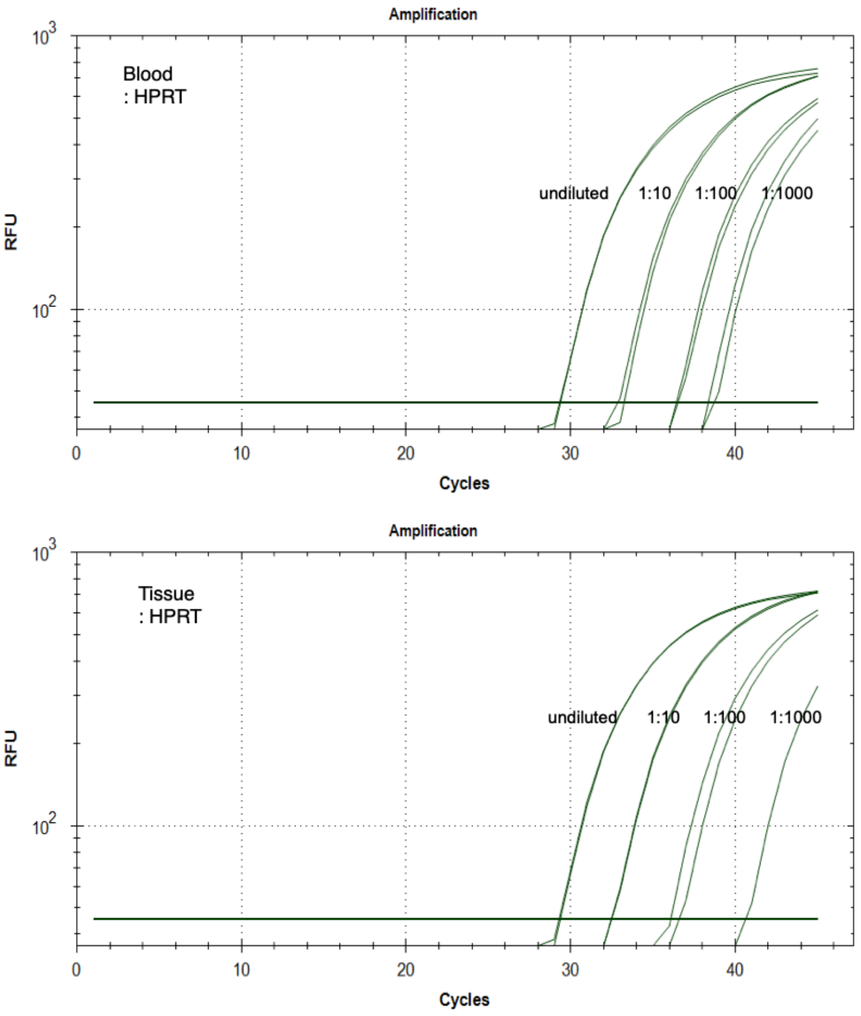

Figure S13. Amplification curves of HPRT detection by using canine peripheral blood and non-neoplastic mammary tissue, undiluted and in 1:10, 1:100 and 1:1000 dilutions.

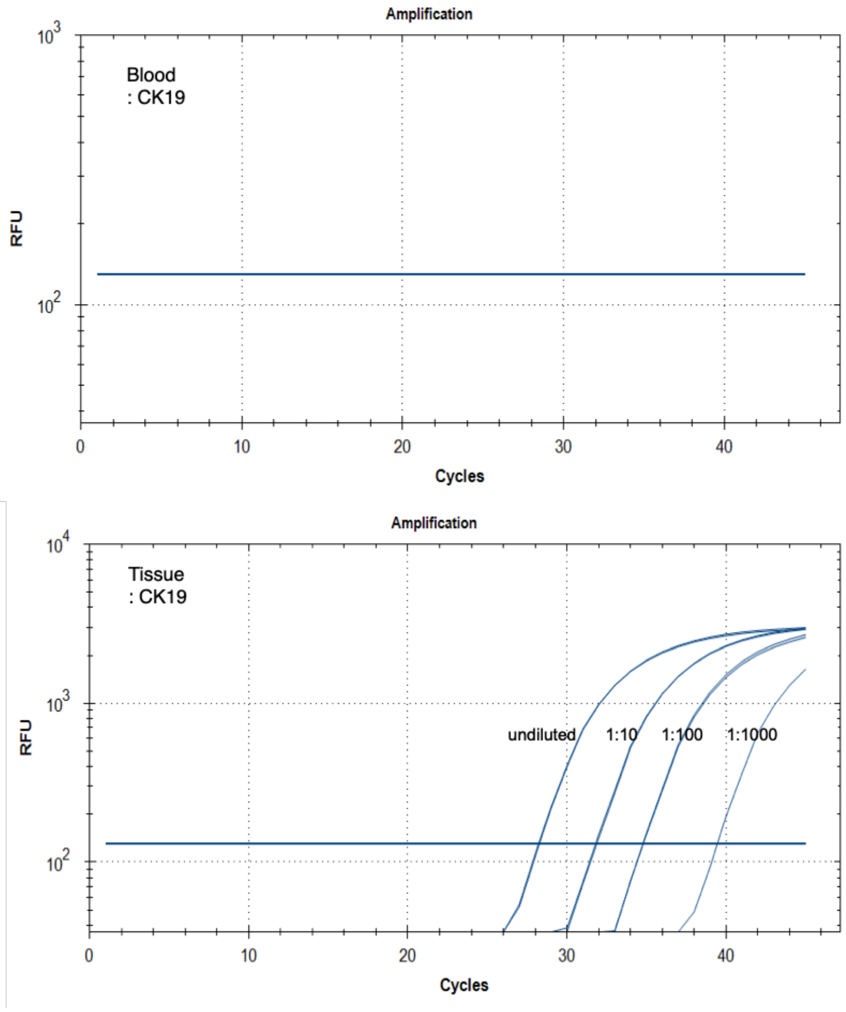

Figure S14. Amplification curves of CK19 detection by using canine peripheral blood and non-neoplastic mammary tissue, undiluted and in 1:10, 1:100 and 1:1000 dilutions.

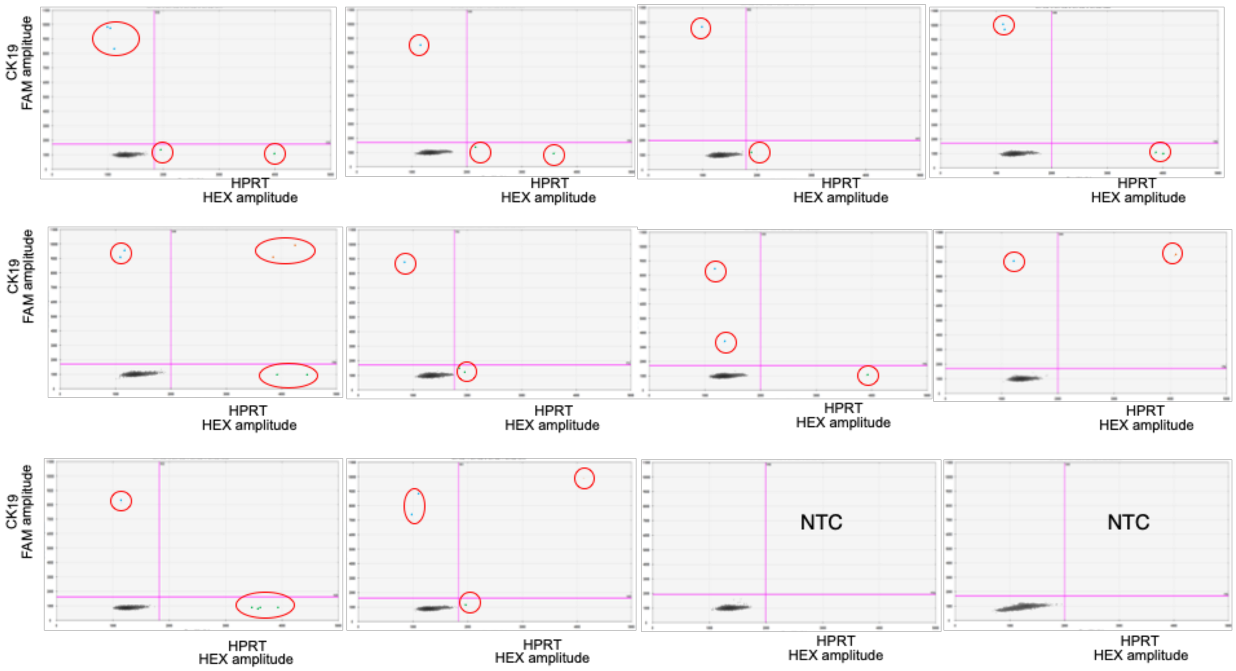

Figure S15. Limit of detection (LOD) of duplex ddPCR in detecting KRT19 (FAM) and HPRT (HEX) (n = 10) and no template control (NTC). The LOD of KRT19 and HPRT assays were 2.16 ± 1.27 and 2.44 ± 1.31 copies/μL, respectively, with 100% detection rates (>95% CI). Blue dots represent CK19, green dots are HPRT, orange dots contain both genes and black dots show when neither gene was detected.

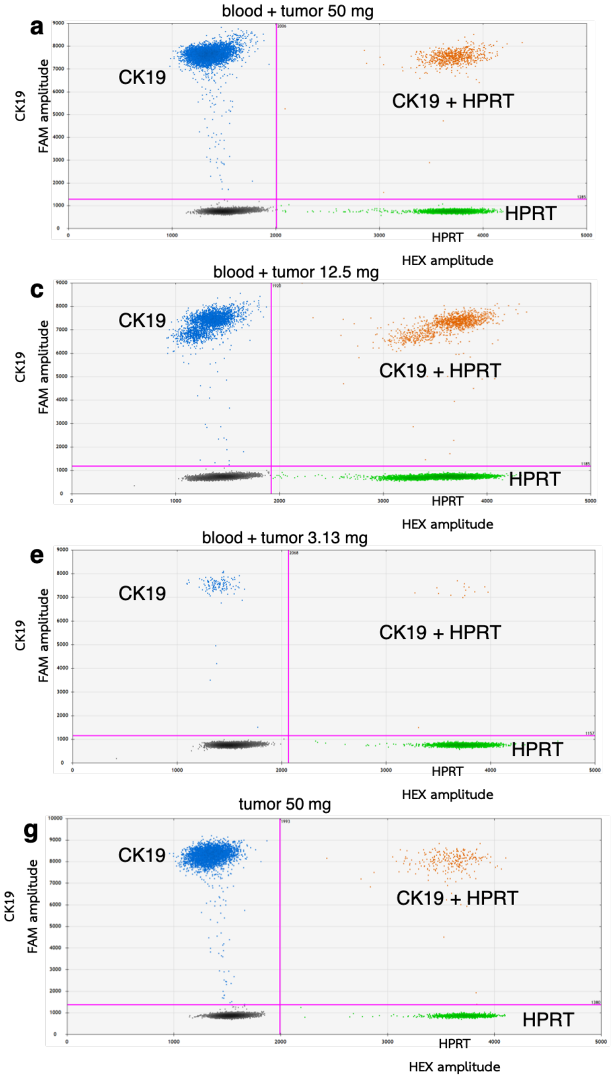

Figure S16. 2D plots of duplex ddPCR results. The assays were tested with the cDNA from spiked samples (50 – 1.56 mg of tumor tissues: a-f) and canine mammary tumor tissue (g). Blue dots represent CK19, green dots are HPRT, orange dots contain both genes and black dots show when neither gene was detected.

Table S1. Digital MIQE checklist.

ITEM TO CHECK	IMPORTANCE	Comments
**EXPERIMENTAL DESIGN**		
Definition of experimental and control groups	E	Included in manuscript
Number within each group	E	Included in manuscript
Assay carried out by core lab or investigator's lab?	D	Core lab
Power analysis	D	Not applicable
**SAMPLE**		
Description	E	Included in manuscript
Volume or mass of sample processed	E	Included in manuscript
Microdissection or macrodissection	E	Not applicable
Processing procedure	E	Not applicable
If frozen - how and how quickly?	E	Not applicable
If fixed - with what, how quickly?	E	Not applicable
Sample storage conditions and duration (especially for FFPE samples)	E	Included in manuscript
**NUCLEIC ACID EXTRACTION**		
Quantification - instrument/method	E	Included in manuscript
Storage conditions: temperature, concentration, duration, buffer	E	Included in manuscript
DNA or RNA quantification	E	Included in manuscript
Quality/integrity-instrument/method; e.g. RIN/RQI and trace or 3':5'	E	Included in manuscript
Template structural information	E	Not applicable
Template modification (digestion, sonication, pre-amplification etc.)	E	Not applicable
Template treatment (initial heating or chemical denaturation)	E	Not applicable
Inhibition dilution or spike;	E	Included in manuscript
DNA contamination assessment of RNA sample	E	Included in manuscript
Details of DNase treatment where performed	E	Included in manuscript
Manufacturer of reagents used and catalogue number	D	Included in manuscript
Storage of nucleic acid: temperature, concentration, duration, buffer	E	Included in manuscript
**REVERSE TRANSCRIPTION (If necessary)**		
cDNA priming method + concentration	E	Included in manuscript
One or two step protocol	E	Included in manuscript (two step)
Amount of RNA used per reaction	E	Included in manuscript
Detailed reaction components and conditions	E	Included in manuscript
RT efficiency	D	Not applicable
Estimated copies measured with and without addition of RT	D	Available on request

Manufacturer of reagents used and catalogue number	D	Included in manuscript
Reaction volume (for two step reverse transcription reaction)	D	Included in manuscript
Storage of cDNA: temperature, concentration, duration, buffer	D	Included in manuscript
**dPCR TARGET INFORMATION**		
Sequence accession number	E	Included in manuscript
Location of amplicon	D	Supplementary
Amplicon length	E	Included in manuscript
In silico specificity screen (BLAST, etc)	E	Included in manuscript
Pseudogenes, retropseudogenes or other homologs?	D	Not applicable
Sequence alignment	D	Supplementary
Secondary structure analysis of amplicon and GC content	D	Included in manuscript
Location of each primer by exon or intron (if applicable)	E	Exon
Where appropriate, which splice variants are targeted?	E	Not applicable
**dPCR OLIGONUCLEOTIDES**		
Primer sequences and/or amplicon context sequence	E	Included in manuscript
RTPrimerDB Identification Number	D	Not applicable
Probe sequences	D	Included in manuscript
Location and identity of any modifications	E	Included in manuscript
Manufacturer of oligonucleotides	D	Included in manuscript
Purification method	D	Desalt for primers and HPLC for probes
**dPCR PROTOCOL**		
Complete reaction conditions	E	Included in manuscript
Reaction volume and amount of RNA/cDNA/DNA	E	Included in manuscript
Primer, (probe), Mg++ and dNTP concentrations	E	Included in manuscript
Polymerase identity and concentration	E	Included in manuscript
Buffer/kit Catalogue No and manufacturer	E	Included in manuscript
Exact chemical constitution of the buffer	D	Not applicable
Additives (SYBR Green I, DMSO, etc.)	E	Not applicable
Plates/tubes Catalogue No and manufacturer	D	Included in manuscript
Complete thermocycling parameters	E	Included in manuscript
Reaction setup	D	Included in manuscript
Gravimetric or volumetric dilutions (manual/robotic)	D	Volumetric dilutions (Manual)
Total PCR reaction volume prepared	D	Included in manuscript
Partition number	E	13000 – 19000

Individual partition volume	E	0.85 μL
Total volume of the partitions measured (effective reaction size)	E	20 μL
Partition volume variance/standard deviation	D	Not applicable
Comprehensive details and appropriate use of controls	E	Included in manuscript
Manufacturer of dPCR instrument	E	Included in manuscript
**dPCR VALIDATION**		
Optimisation data for the assay	D	Included in manuscript
Specificity (when measuring rare mutations, pathogen sequences etc.)	E	Included in manuscript
Limit of detection of calibration control	D	Included in manuscript
If multiplexing, comparison with singleplex assays	E	Not applicable
**DATA ANALYSIS**		
Average copies per partition (λ or equivalent)	E	Average 0.09
dPCR analysis program (source, version)	E	Included in manuscript
Outlier identification and disposition	E	Not applicable
Results of NTCs	E	Supplementary
Examples of positive(s) and negative experimental results as supplemental data	E	Supplementary
Where appropriate, justification of number and choice of reference genes	E	Included in manuscript
Where appropriate, description of normalisation method	E	Not applicable
Number and concordance of biological replicates	D	Not applicable
Number and stage (RT or qPCR) of technical replicates	E	Included in manuscript
Repeatability (intra-assay variation)	E	Included in manuscript
Reproducibility (inter-assay/user/lab etc. variation)	D	Not applicable
Experimental variance or confidence interval	E	Included in manuscript
Statistical methods used for analysis	E	Included in manuscript
Data submission using RDML	D	Not applicable

All essential information (E) must be submitted with the manuscript. Desirable information (D) should be submitted if possible.
